# Sexual health interventions for treating sexual dysfunction in women with female genital mutilation: A systematic review

**DOI:** 10.1002/ijgo.70761

**Published:** 2026-01-26

**Authors:** Uduak Okomo, Chioma Oringanje, Miriam Nwaneri‐Ogugbue, Elizabeth Inyang, Babatunde Okusanya, Petra‐Favour Okoh, Mavis Otonkue, Martin Meremikwu

**Affiliations:** ^1^ Clinical Research Department and MARCH Centre London School of Hygiene & Tropical Medicine London UK; ^2^ Vaccines and Immunity Theme MRC Unit The Gambia at London School of Hygiene & Tropical Medicine Fajara The Gambia; ^3^ Cochrane Nigeria, Calabar Institute of Tropical Diseases Research and Prevention, University of Calabar Teaching Hospital Calabar Nigeria; ^4^ Biology Department Xavier University Cincinnati OH USA; ^5^ Public Health, School of Health and Wellbeing University of Glasgow Glasgow UK; ^6^ College of Medicine University of Lagos/Lagos University Teaching Hospital Idi‐Araba Lagos Nigeria

**Keywords:** female genital mutilation, female sexual dysfunction, Female Sexual Function Index, mechanical devices, psychological therapy, sexual counseling, sexual therapy

## Abstract

**Background:**

Female sexual dysfunction (FSD), characterized by persistent problems with desire, arousal, orgasm, or pain, can occur in women with any type of female genital mutilation (FGM) as a result of anatomical changes, pain, or psychological trauma.

**Objectives:**

To systematically review the evidence on the effects of non‐surgical interventions, including sexual counseling, mechanical devices, and lubricants, on the sexual function in women living with FGM.

**Search Strategy:**

A comprehensive search was conducted in CINAHL Plus, IRIS, MEDLINE (Ovid), PsycINFO (EBSCOhost), SCOPUS, and Web of Science from inception to November 2025. Reference lists were hand‐searched and study authors contacted for additional data.

**Selection Criteria:**

Studies were eligible if they involved women with any type of FGM who received non‐surgical interventions for FSD.

**Data Collection and Analysis:**

One controlled trial met the inclusion criteria. Data were extracted independently by two reviewers, and the certainty of the evidence was assessed using the GRADE approach.

**Main Results:**

In women with Type I FGM, use of the FDA‐approved Eros‐Clitoral Therapy Device (CTD) combined with psychotherapy led to statistically significant improvements across all domains of the Female Sexual Function Index compared with psychotherapy alone. In the control group, only orgasm scores improved.

**Conclusions:**

Evidence on non‐surgical interventions for FSD in women with FGM is extremely limited and based solely on a small single trial in women with Type I FGM. Although Eros‐CTD shows promise, findings cannot be generalized to other FGM types, and data on safety and contraindications are lacking. Further research is needed across diverse populations and FGM types to inform practice and policy.

## 1 INTRODUCTION

### 1.1 Background

WHO defined female genital mutilation (FGM) as “all procedures which intentionally alter or damage the external female genital organs for non‐medical reasons, and which have no benefit for the health of young girls and women”.^1^ FGM remains prevalent in parts of Africa, the Middle East, Asia, South America, and the Pacific.^2^ With increasing global migration, the number of girls and women affected by FGM outside these regions has risen, particularly among certain minority groups and immigrant communities in Europe, North America, and Australia. The practice is often motivated by cultural, religious, and social beliefs surrounding femininity, marriageability, and sexual morality, and is closely associated with ideas of premarital virginity and marital fidelity.^3^, ^4^, ^5^

FGM is classified into four types: Type I (clitoridectomy) involves partial or total removal of the clitoral glans and prepuce; Type II (excision) involves partial or total removal of the clitoris and labia minora with or without excision of the labia majora; Type III (infibulation) involves narrowing of the vaginal orifice through labia apposition, with or without clitoral removal; and Type IV includes other harmful procedures on the external genitalia, such as piercing, pricking, stretching, scraping, or cauterization.^6^

Sexual health is a state of physical, emotional, mental, and social well‐being in relation to sexuality, encompassing freedom from disease and dysfunction as well as the ability to experience safe, pleasurable, and consensual sexual relationships free from coercion, discrimination, and violence.^7^ A recent systematic review found that FGM is associated with female sexual dysfunction (FSD) and other adverse sexual health outcomes.^8^ FSD includes persistent or recurrent problems with sexual desire, arousal, orgasm, or pain during intercourse. It results from complex interactions involving the clitoris, vagina, labia minora, vestibular bulbs, pelvic floor muscles, and uterus. The clitoris, a highly sensitive organ essential to female sexual response, is often affected in FGM.^9^ FSD among women with FGM is multifactorial, resulting from anatomical distortion, chronic urogenital pain, and psychological trauma, particularly in those with scarring.^10^ Psychological and social‐interpersonal factors further contribute to dysfunction, with many women reporting distressing memories, sexual anxiety, reduced lubrication, increased pain, decreased sexual satisfaction and desire, and anorgasmia.^2^, ^11^, ^12^, ^13^, ^14^, ^15^, ^16^

The Female Sexual Function Index (FSFI) is the most widely used tool to evaluate six key domains of sexual function: desire, arousal, lubrication, orgasm, satisfaction, and pain.^17^ Studies consistently show lower scores among women who have undergone FGM.^13^, ^18^, ^19^

Effective management of FSD requires a multidisciplinary approach. A systematic review identified multiple treatment options, including hormonal therapy, lubricants, behavioral and psychotherapeutic interventions, and mechanical devices.^20^, ^21^ Physiotherapy, particularly pelvic floor muscle training with biofeedback, improve sexual pain and function.^22^ Among women with FGM, surgical procedures such as deinfibulation and clitoral reconstruction, can enhance sexual satisfaction, although results vary.^23^, ^24^ Counseling addresses the psychological and cultural aspects of sexuality in women with FGM; however, a 2015 review found no clear evidence of its effectiveness in managing or preventing FSD.^25^

Recognizing the need to update guidelines for managing health complications arising from FGM, WHO committed to revising its protocols in 2023.

### 1.2 Objective

The aim of this systematic review was to update and expand our 2015 review by evaluating the effects of non‐surgical sexual health interventions—including counseling, psychological therapies, and physical therapy—on sexual function and broader sexual health outcomes, such as body image, psychosexual functioning, and overall quality of life in women with FGM.

## 2 METHODS

The review was conducted in line with the Cochrane Handbook for Systematic Reviews of Interventions version 5.1.0,^26^ and the findings were reported in accordance with the Preferred Reporting Items for Systematic Reviews and Meta‐Analyses (PRISMA) guidelines.^27^, ^28^ The protocol of this updated review was registered with the International Prospective Register of Systematic Reviews (PROSPERO; http://www.crd.york.ac.uk/PROSPERO/) (ID: CRD42023435048).

### 2.1 Search strategy

A systematic search for published and gray literature was carried out in the following electronic databases from inception to May 26, 2023: CINAHL Plus, IRIS, MEDLINE (Ovid), PsychINFO (EBSCOhost), SCOPUS, and Web of Science. An additional search was conducted from January 2023 to November 2025. The search terms consisted of keywords specific to the database and text words for FGM, FSD, and various sexual health interventions (Tables S1–S6). We did not apply any limits on language and geographical location. The reference lists of included studies and relevant systematic reviews were checked to identify articles that could have been missed in the electronic searches.

### 2.2 Eligibility criteria

This review considered studies conducted among girls and women who had undergone FGM and were diagnosed with FSD. We prioritized randomized controlled trials (RCTs). Where these were unavailable, the plan was to consider other designs if they had comparable arms and at least one arm included a sexual health intervention. Eligible studies compared various sexual health interventions, such as counseling/psychotherapy and physical therapy, with no treatment. In addition, we considered studies that compared counseling/psychotherapy or physical therapy with no treatment, other forms of sexual therapy, or pharmacological interventions, such as the use of genital lubricants or hormonal therapy. Studies evaluating a combination of counseling/psychotherapy and physical therapy to other types of sexual therapy or any other single intervention were also considered. For this review, sexual health interventions were defined as all pharmacological and non‐pharmacological (excluding surgical) interventions aim at treating sexual dysfunction in women who have undergone any form of FGM.

We defined “sexual counseling” as any service provided by a qualified health professional or trained individual, such as a sex therapist, intended to address the sexual concerns of women affected by FGM. These interventions include assessment, support, and targeted advice regarding psychosexual and sexual issues directly related to FGM. For this review, the following operational definition, adapted from similar interventions used for women with non‐FGM‐associated sexual dysfunction^29^, ^30^ was used to describe physical therapy interventions for women with FGM‐related sexual dysfunction to be “any treatment provided by a qualified professional within the physical therapy scope of practice, including a physical therapist, to improve sexual function and satisfaction”. These interventions may include electrical stimulation, electrotherapy, extracorporeal shockwave therapy, biofeedback, muscle retraining exercises, and pelvic floor manual therapy.

Primary outcomes included improvement in psychosexual functioning, measured by the FSFI score or other validated instruments, improved sexual health outcomes, reduction in acute or chronic vulvar or clitoral pain, and improvement in symptoms of vulvodynia and/or clitoral pain. Secondary outcomes included an improvement in sexual pleasure, quality of life, sexual function assessed by other measures, male sexual function (BSFI score), body image, and any reported adverse events, such as worsening symptoms, marital discord, intimate partner violence, decreased self‐confidence or self‐esteem related to body image, or increased body insecurities.

### 2.3 Selection of studies

**FIGURE 1:**
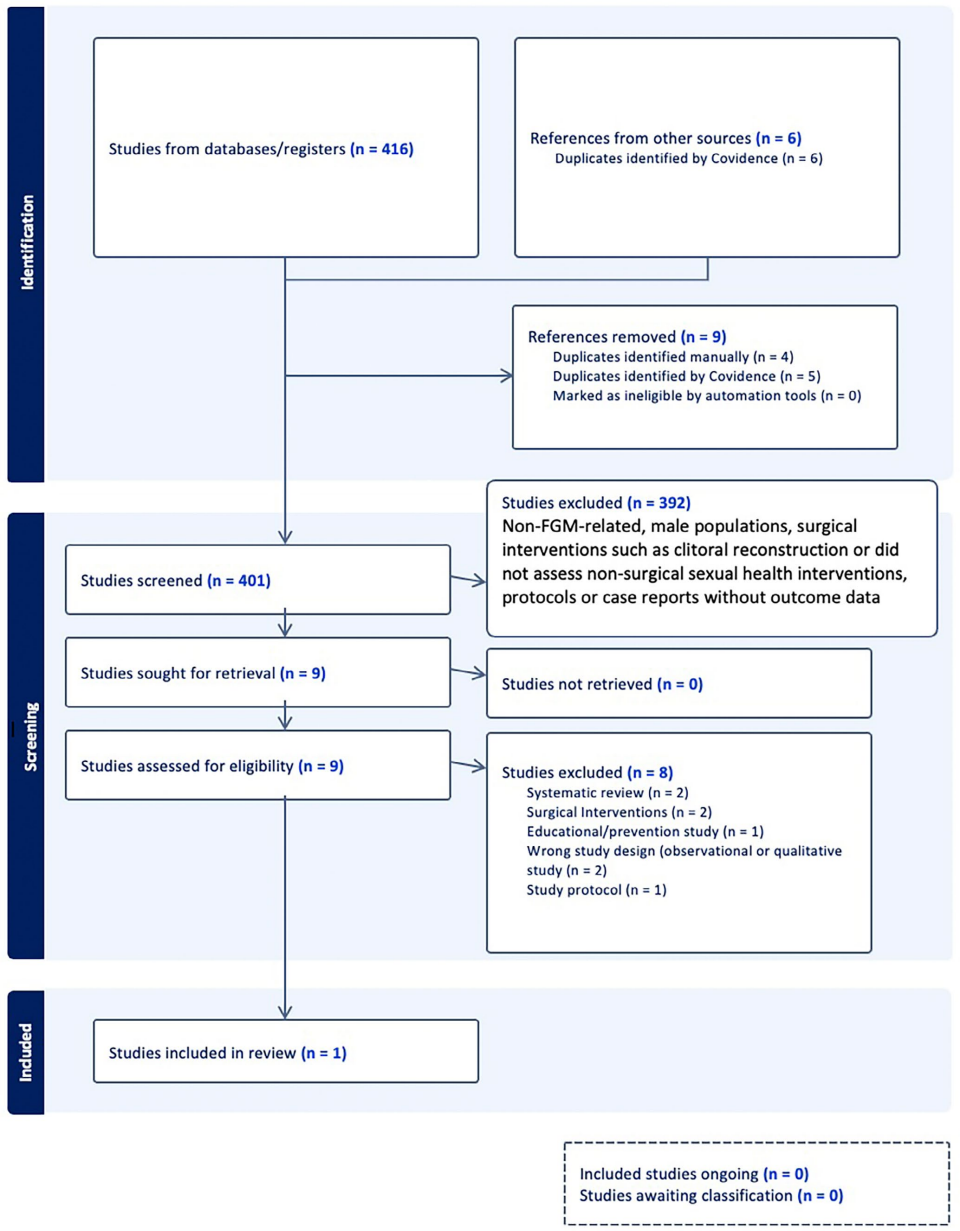
PRISMA flow diagram.

The literature search results were uploaded into the Covidence systematic review software and checked for duplicates. After removing duplicates, two authors independently screened all titles and abstracts for inclusion. To ensure consistency and minimize bias, a third reviewer assessed a random 20% sample of excluded studies for quality control. The 20% proportion was selected to ensure methodological rigor while maintaining feasibility given the large number of records screened. Any disagreements were resolved through discussion with a senior reviewer. Full‐text reports were obtained for titles that met the inclusion criteria. Any discrepancies were resolved through discussion among the review team. Details of the search results are shown in a PRISMA flowchart (Figure 1).

### 2.4 Data extraction

Data were extracted using a specially developed and piloted form by two independent reviewers. The following information was collected: population characteristics (place, race, occupation, religion, education, social capital, socioeconomic status, type of FGM, type and severity of complication, and associated physical or psychosocial morbidities); details about the intervention and comparison groups; and the outcomes assessed. Where reported, the plan was to extract information regarding adverse outcomes, including but not limited to worsening of symptoms, marital disharmony, and intimate partner violence.

For each outcome, the number of participants randomized or included in the study, and the number analyzed in each arm were extracted. For dichotomous outcomes, the number of participants experiencing the event and the total assessed in each arm were recorded. For continuous outcomes, arithmetic means, standard deviations, and the numbers evaluated in each arm were extracted.

Data were extracted from the tables. In cases where the text and table results are inconsistent, table data were prioritized for accuracy. The lead author was contacted for additional data and further clarification.

### 2.5 Assessment of risk of bias

The risk of bias of the included study was independently assessed by two reviewers using the Cochrane Risk of Bias tool for RCTs.^31^ We assessed the balance between comparator groups at baseline for key prognostic or confounding factors. For each arm, we assessed eight components: random sequence generation; allocation concealment; blinding (performance and detection bias); baseline outcome measurement; similarity in baseline characteristics; incomplete outcome data; selective outcome reporting; and other biases. Risk of bias was classified as “yes”, “no”, and “unclear” to indicate a low, high, or unclear risk of bias and results are presented in the “Risk of bias” tables and summary. We attempted to contact the study authors when required to clarify unclear information.

### 2.6 Data synthesis

We included only one comparative effectiveness study (RCT). Meta‐analysis was therefore impossible, and we present a narrative summary of the data based on SWiM guidelines.^32^ We assessed the certainty of the evidence (CoE) using the Grading of Recommendations Assessment Development and Evaluation (GRADE) Working group methodology and GRADEpro.^33^ We appraised the certainty of the evidence for each outcome against five criteria: risk of bias (an appraisal of the overall risk of bias for trials contributing to the outcome); inconsistency (an evaluation of explained and unexplained heterogeneity); indirectness (an appraisal of how directly the included trials address the review question); imprecision (an assessment of the statistical precision of the result); and publication bias (an assessment of the risk of publication bias).

## 3 RESULTS

### 3.1 Study selection

We identified 416 potentially relevant records using the search strategies. We excluded 15 duplicates. After reviewing the titles and abstracts, we excluded 392 irrelevant records. Studies were excluded if they were not FGM‐related, involved male populations, focused on surgical interventions, did not assess non‐surgical sexual health interventions, were protocols or case reports without outcome data, or were descriptive, illustrative, or qualitative‐only studies. We obtained the full‐text reports of the nine eligible studies for screening. Eight studies were excluded, with reasons for exclusion detailed in Table 1. Only one study met the inclusion criteria,^34^ as shown in Figure 1.

**TABLE 1:** Characteristics of excluded studies.

Study ID^a^	Reason for exclusion
Abdulcadir (2017)	Case report; primarily surgical intervention, non‐surgical care not systematically evaluated
Beltran (2015)	Study protocol
Elnashar (2007)	Observational prevalence study; no non‐surgical intervention tested
Ezebialu (2017)	Systematic review, no empirical data; review found no studies on non‐surgical interventions for pain in FGM
Foldès (2013)	Review of surgical genital procedures; non‐surgical interventions only mentioned, not evaluated
Mahgoub (2019)	Educational prevention study; does not address treatment of sexual dysfunction in women with FGM
Okomo (2017)	Systematic review, no empirical data; review found no studies on counseling or lubricants for sexual dysfunction in FGM
Villani (2019)	Wrong study type; qualitative evidence on the mechanisms and outcomes of sexual counseling

Abbreviation: FGM, female genital mutilation.

^a^ References to excluded studies can be found in File S2.

### 3.2 Study characteristics

Details of the included study are shown in Table 2. The study was a randomized double‐blind trial conducted at an outpatient gynecology clinic in Cairo, Egypt.^34^ Participants were randomly assigned to one of two equal groups using index cards in sealed envelopes. The study participants comprised 80 married women aged 20–45 years who had undergone Type 1 FGM (clitoridectomy) and had been diagnosed with sexual dysfunction in more than one sexual domain (arousal disorder, orgasm disorder, or both). Each participant underwent a comprehensive medical history assessment and a pelvic examination. Women with metastases, bladder or bowel disorders, major disease complications, a history of female sexual disease or sexual assault, or those using antidepressant medications were excluded.

**TABLE 2:** Characteristics of included studies.

Method	RCT; before and after comparison within each of the study arms
Participants	80 married women aged 20–45 years who experienced sexual dysfunction in more than one sexual domain (arousal disorder, orgasm disorder, or both); have Type I FGM (clitoridectomy); all the participants had sexual desire, were comfortable with the ideas of self‐stimulation and psychosexual support, and were medically stable Participants were referred from the gynecology clinic of the Faculty of Medicine, Suez University, to the outpatient clinic of the Faculty of Physical Therapy, Badr University, located in Cairo, Egypt between September 2021 and December 2021 Exclusion: Women with metastatic disease, severe bladder or bowel disorder, and significant comorbidities; a history of female sexual disease or sexual trauma or abuse; women undergoing antidepressant therapy during the study period
Intervention	Experimental group: Traditional psychosexual education (or sex therapy) sessions plus the Eros‐CTD Control: Traditional psychosexual education (or sex therapy) only The Eros‐CTD is a small, hand‐held device fitted with a removable, replaceable small plastic cup used as a natural way to initiate female sexual response Psychosexual (behavioral) support involved suggestion of strategies to improve the couple's emotional connection and communication. Each couple was encouraged to focus on the strengths and weaknesses of their relationship. Patients and their partners were educated the on the stages of sexual arousal before orgasm. Homework assignments were given for the couple to practice skills, such as turning the idea of sexual obligation into pleasure, learning to focus on sensations rather than anxieties, and communicating openly with their partner. Patients were encouraged to gradually follow certain steps: first, to stroke the full body outside the genital areas; second, to learn how to change between active and passive positions and massage the body and genital areas using hand stimulation; and third, the woman inserted the penis into the vagina and the couple experimented with different sex positions. The participants applied these steps and returned weekly with their feedback to the therapist
Outcome	Change in the FSFI score from baseline sexual function at 3 months. The following domains were assessed: Pain, Satisfaction, Orgasm, Arousal, and Lubrication
Notes	The authors use the terms psychosexual (behavioral) support, psychosexual therapy, psychosexual education, and sex therapy interchangeably Follow‐up: 3 months Loss to follow‐up: None

Abbreviations: CTD, clitoral therapy device; FGM, female genital mutilation; FSFI, Female Sexual Function Index; RCT, randomized controlled trial.

The study compared the effectiveness of using traditional psychosexual education plus the Eros Clitoral Therapy Device (CTD) versus traditional psychosexual education alone for treating sexual dysfunction. The Eros‐CTD is a non‐pharmacological technique certified by the US Food and Drug Administration (FDA) for promoting female sexual function by improving orgasm quality (i.e., the regularity of orgasm by direct clitoral stimulation). It is a small, hand‐held device fitted with a removable, replaceable small plastic cup and promotes clitoris engorgement, causing sensory nerve‐ending stimulation. Upon enrolment, the women in the intervention group received instructions on using the device and were encouraged to try it out at the clinic. They were then provided with the device to use at home, either independently or with a partner, for four sessions per week over 3 months. The recommended usage was 5–15 min of continuous or 30 min of intermittent application. Psychoeducation was provided and included educating the patients and their partners on the stages of sexual arousal before orgasm and giving them tips and techniques to be applied at home. The participants returned weekly with their feedback to the therapist. Before treatment, each participant was requested to fill out the Arabic FSFI questionnaire. After 3 months of regular treatment, participants completed the questionnaire again. The study reported the following domains using the FSFI: pain, satisfaction, orgasm, arousal, lubrication, and desire.

### 3.3 Risk of bias of the included study

**FIGURE 2:**
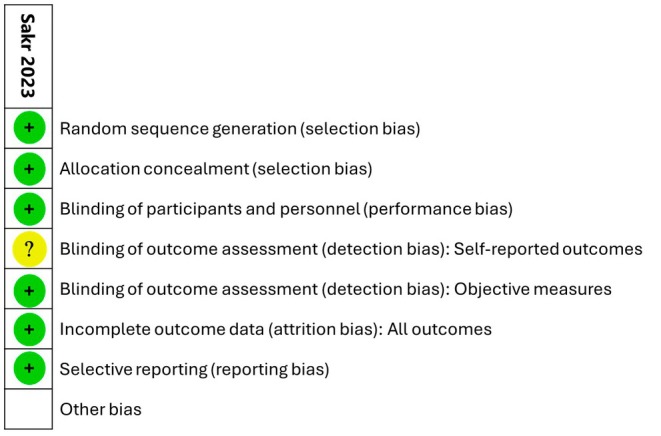
Risk of bias summary.

The risk of bias assessment for the included study is presented in Figure 2, with detailed information provided in Table 3. Overall, the study was assessed as having a low risk of bias. The study employed a randomized design using a computer‐generated sequence prepared by an independent researcher, with allocation concealed in a securely sealed opaque envelopes, minimizing bias in random sequence generation and allocation concealment. This study was a double‐blinded RCT, with group assignment performed by an independent researcher unaware of baseline assessments. Although the blinding of outcome assessors remains uncertain, the outcomes were measured using the well‐established and validated FSFI questionnaire, reducing performance and detection bias due. Although the risk of detection bias due to self‐reported outcomes is unclear, the absence of loss to follow‐up suggests a low risk of bias for incomplete outcome data. In addition, the study's adherence to reporting prespecified outcomes from its clinical trial registry supports a low risk of selective reporting bias.

**TABLE 3:** Summary of findings: Psychotherapy education (or sex therapy) plus Eros‐CTD versus psychotherapy education (or sex therapy) alone.

Certainty assessment							Impact	Certainty
No. of studies	Study design	Risk of bias	Inconsistency	Indirectness	Imprecision	Other considerations		
**Pain domain**								
1	RCTs	Not serious	Not serious	Serious^a^	Serious^b^	None	Between group: Median post‐treatment score was 3.6 (IQR 2.4–3.6) and 0 (IQR 0–1.2) in the experimental and control groups, respectively (*P* < 0.001). Within group: For the experimental group (*n* = 40) the median pre‐treatment score was 0 (IQR 0–1.2) versus post‐treatment score of 3.6 (IQR 2.4–3.6) (*P* < 0.001). In the control group (*n* = 40), the median pre‐treatment score was 1.2 (IQR 0–1.2) versus the post‐treatment score of 0 (IQR 0–1.2) (*P* < 0.05)	⨁⨁◯◯ Low
**Satisfaction domain**								
1	RCTs	Not serious	Not serious	Serious^a^	Serious^b^	None	Between group: Median post‐treatment score was 3.6 (IQR 2.4–3.6) and 0 (IQR 0–1.2) in the experimental and control groups, respectively (*P* < 0.001). Within group: For the experimental group (*n* = 40) the median pre‐treatment score was 0 (IQR 0–1.2) versus post‐treatment score of 3.6 (IQR 2.4–3.6) (*P* < 0.001) In the control group (*n* = 40), the median pre‐treatment score was 0.6 (IQR 0–1.2) versus post‐treatment score of 0 (IQR 0–1.2) (*P* = 0.46)	⨁⨁◯◯ Low
**Orgasm domain**								
1	RCTs	Not serious	Not serious	Serious^a^	Serious^b^	None	Between group: Median post‐treatment score was 1.2 (IQR 1.2–3.6) and 0 (IQR 0–0) in the experimental and control groups, respectively (*P* < 0.001) Within group: For the experimental group (*n* = 40), the median pre‐treatment score was 0 (IQR 0–0) versus post‐treatment score of 1.2 (IQR 1.2–3.6) (*P* < 0.001) In the control group (*n* = 40), the median pre‐treatment score was 0 (0–0) versus post‐treatment score of 0 (IQR 0–0) (*P* = 0.16)	⨁⨁◯◯ Low
**Arousal domain**								
1	RCTs	Not serious	Not serious	Serious^a^	Serious^b^	None	Between group: Median post‐treatment score was 3.6 (IQR 2.4–3.6) and 0 (IQR 0–1.2) in the experimental and control groups, respectively (*P* < 0.001). Within group: For the experimental group (*n* = 40), the median pre‐treatment score was 0 (IQR 0–1.2) versus post‐treatment score of 3.6 (IQR 2.4–3.6) (*P* < 0.001) In the control group (*n* = 40), the median pre‐treatment score was 1.2 (IQR 0–1.2) versus post‐treatment score of 0 (IQR 0–1.2) (*P* < 0.05)	⨁⨁◯◯ Low
**Lubrication domain**								
1	RCTs	Not serious	Not serious	Serious^a^	Serious^b^	None	Between group: Median post‐treatment score was 4.8 (IQR 3.6–4.8) and 0 (IQR 0–1.2) in the experimental and control groups, respectively (*P* < 0.001) Within group: For the experimental group (*n* = 40), the median pre‐treatment score was 1.2 (IQR 0–1.2) versus post‐treatment score of 4.8 (IQR 3.6–4.8) (*P* < 0.001) In the control group (*n* = 40), the median pre‐treatment score was 1.2 (IQR 0–1.2) versus post‐treatment score of 0 (IQR 0–1.2) (*P* < 0.05)	⨁⨁◯◯ Low
**Desire domain**								
1	RCTs	Not serious	Not serious	Serious^a^	Serious^b^	None	Between group: Median post‐treatment score was 4.8 (IQR 3.6–4.8) and 1.2 (IQR 1.2–2.4) in the experimental and control groups, respectively (*P* < 0.001) Within group: For the experimental group (*n* = 40), the median pre‐treatment score was 1.2 (IQR 1.2–2.4) versus post‐treatment score of 4.8 (IQR 3.6–4.8) (*P* < 0.001) In the control group (*n* = 40), the median pre‐treatment score was 1.8 (IQR 1.2–2.4) versus post‐treatment score of 1.2 (IQR 1.2–2.4) (*P* < 0.05)	⨁⨁◯◯ Low

*Note*: Question: Psychosexual education (or sex therapy) plus Eros‐clitoral therapy device compared to psychosexual education (or sex therapy) alone.

Abbreviations: CTD, clitoral therapy device; IQR, interquartile range; RCT, randomized controlled trial.

^a^ Small sample size.

^b^ Study assessed a CTD in addition to psychosexual education.

### 3.4 Synthesis of results

The study reported no significant pre‐treatment differences between groups across all FSFI domains. At 3 months after treatment, the experimental group showed a significant increase in all domains compared to the control group. Specifically, median post‐treatment scores in the experimental versus controls groups were as follows: Pain 3.6 (interquartile range [IQR] 2.4–3.6) and 0 (IQR 0–1.2); Satisfaction 3.6 (IQR 2.4–3.6) versus 0 (IQR 0–1.2); Orgasm 1.2 (IQR 1.2–3.6) versus 0 (IQR 0–0); Arousal 3.6 (IQR 2.4–3.6) versus 0 (IQR 0–1.2); Lubrication 4.8 (IQR 3.6–4.8) versus 0 (IQR 0–1.2); and Desire 4.8 (IQR 3.6–4.8) versus 1.2 (IQR 1.2–2.4) (all *P* < 0.001).

In the experimental group, four of the six FSFI domains improved significantly from pre to post treatment (*P* < 0.5). The authors also report a similar improvement in four of the six FSFI domains for the control group in the written summary. However, the table data show no improvement in five of the six domains in the control group, except for orgasm, which reports minimal change or worsening of scores after treatment. Attempts to clarify these discrepancies with the corresponding author were unsuccessful.

## 4 DISCUSSION

### 4.1 Comparison with existing literature

This systematic review found that mechanical Eros‐CTD combined with traditional psychosexual behavioral support compared to no therapy (within‐group comparison) or compared to psychosexual behavioral support alone (between‐group comparison) improved sexual function among women with type I FGM. Sexual function worsened in most domains among women who received psychotherapy support alone, except for orgasm.

Mechanical devices are non‐invasive and non‐pharmacological options for treating FSD, particularly arousal and orgasm disorders. They can be broadly categorized as mechanical vibrators or clitoral vacuum engorgement devices, with distinct mechanisms of action; the Eros‐CTD falls into the latter category.^35^ Mechanical vibrators have been available for decades and are used to treat primary and secondary anorgasmia, often in conjunction with psychological counseling.

There are a few small non‐randomized studies that examined the effectiveness of the Eros‐CTD among women with and without FSD. Billups et al.^36^ reported an improvement in genital/clitoral sensation, vaginal lubrication, orgasmic ability, and overall sexual satisfaction among women with FSD. Similar results were observed in a periodic survey of 19 women using the device over 6 weeks.^37^ Other studies have also reported statistically significant improvements in all domains of sexual function tested, as well as reduced dyspareunia after using clitoral therapy devices for 3 months among postmenopausal women, cervical cancer patients, women with spinal cord injury or multiple sclerosis, and diabetic women.^38^, ^39^, ^40^

The included study did not assess risks associated with the use of Eros‐CTD, but reported selecting this device over other devices based on US FDA safety approval. Only one prior study evaluated its safety in women without FGM and reported no adverse effects such as skin irritation, hypersensitivity, infection, hematoma, clinical trauma, compromise of skin integrity, or allergic response to the device material.^36^ However, these findings cannot be extrapolated to establish effectiveness, safety, or acceptability of Eros‐CTD in women with FGM, as physiological and psychosocial contexts differ significantly.

### 4.2 Certainty of the evidence

The certainty of evidence of the included study was rated as moderate. Risk of bias was generally low risk, except for unclear blinding of outcome assessors. Outcomes were self‐reported using the FSFI questionnaire, which has a validated cutoff score of 26.55 for differentiating women with and without sexual dysfunction.^41^

### 4.3 Strengths and limitations

This review followed Cochrane‐recommended systematic review methodology, including duplicate screening, data extraction, and risk of bias assessment.^42^ Comprehensive searches were conducted without language restrictions to minimize publication bias. However, several important limitations must be emphasized. The findings are limited in generalizability, as the study included only women with Type I FGM in Egypt and cannot be extended to women with other types of FGM or to different cultural, geographical, or clinical settings. The evidence base is also extremely limited, with only one study meeting the inclusion criteria, reflecting a critical gap in the literature rather than a methodological limitation of this review. Furthermore, the study did not report on adverse effects or long‐term outcomes, leaving evidence on safety and acceptability largely unknown; only a single prior study in women without FGM reported no adverse effects.

The focus on a single intervention, the Eros‐CTD, further restricts applicability, as data on other mechanical or non‐mechanical interventions for women with FGM are lacking. Taken together, these limitations underscore the need for cautious interpretation; the review does not provide definitive evidence for clinical effectiveness but rather highlights urgent gaps in research.

## 5 CONCLUSION

Future research should include women with diverse FGM types and from varied cultural and geographical contexts to enhance generalizability. Studies should evaluate safety, acceptability, feasibility, cost‐effectiveness, and long‐term outcomes, and explore a broader range of interventions, including psychosocial, behavioral, and multimodal approaches. Importantly, research should be conducted within culturally sensitive frameworks that account for the unique physical, psychological, social, and religious contexts of women affected by FGM.

## AUTHOR CONTRIBUTIONS

UO and MM developed the protocol. MN, EI, BO, PO, and MO screened search results for eligibility. CO and MM conducted the risk of bias assessment. UO and CO wrote the draft versions of the review. All authors read the draft versions of the review and approved the final version.

## FUNDING INFORMATION

This work received funding from the Government of Norway and the UNDP‐UNFPA‐UNICEF‐WHO‐World Bank Special Programme of Research, Development, and Research Training in Human Reproduction (HRP), a cosponsored programme executed by the World Health Organization (WHO).

## CONFLICT OF INTEREST STATEMENT

The authors have no conflicts of interest.

## Supporting information


File S1.

File S2.

## Data Availability

Data sharing is not applicable to this article as no new data were created or analyzed in this study.
