# Supplementary material for: Sexual health interventions for treating sexual dysfunction in women with female genital mutilation: A systematic review
Source: Int J Gynaecol Obstet. 2026 Jan 26;172(Suppl 1):9–19. doi: 10.1002/ijgo.70761 (PMC12833628; doi:10.1002/ijgo.70761)
Supplement: Supplementary file 1 — File S1. [file IJGO-172-9-s001.docx]

**Table S1: CINAHL Search strategies and output**

| S18 | S14 AND S17 |
| --- | --- |
| S17 | S15 OR S16 |
| S16 | (MH "Female Genital Mutilation") |
| S15 | (female or girl or girls or women or woman) N3 (circumcis* or "genital cut*" or "genital mutilat*") |
| S14 | S1 OR S2 OR S3 OR S4 OR S5 OR S6 OR S7 OR S8 OR S9 OR S10 OR S11 OR S12 OR S13 |
| S13 | "residential treatment" or "rewind technique" or "sensory feedback" or "stress manag*" or "transactional analysis" or yoga |
| S12 | imagery or logotherapy or meditation or mindfulness or "motivational interview*" or mozart* or music* or neurofeedback or nonpharmacol* or "non-pharmacol*" or "operant conditioning" or pranayama or "progressive muscle relax*" or psychodrama or "psychological adaptation" or "psychological feedback" or psychotherap* |
| S11 | (desensiti?ation N2 psychol*) or "emotional freedom tapping" or (feedback N2 psychol*) or flooding or "free association" or hypnosis or hypnotherapy |
| S10 | catharsis or "classical conditioning" or "compassionate mind train*" or (conditioning N2 psychol*) or counsel#ing or "crisis intervention" |
| S9 | abreaction or aromatherap* or asanas or "autogenic train*" or "behav* modification" or bibliotherap* or (biofeedback N2 psychol*) |
| S8 | relax* W3 (therap* or intervention or treat*) |
| S7 | ("patient cent*" or "person cent*" or play or psychoanalytic or "rational emotive" or reality or self or sleep or socioenvironmental or "socio-environmental" or stigma or suggestion or systemic or systems or "therapeutic community") W2 (therap* or treatment or train* or retrain* or rehabilitat* or adapt* or intervention or manag*) |
| S6 | ("Human Givens" or humanistic or implosive or Interpersonal or language or marital or massage or memory or mentali?ation or milieu or music or narrative or nondirective or "non-directive") W2 (therap* or treatment or train* or retrain* or rehabilitat* or adapt* or intervention or manag*) |
| S5 | ("client cent*" or cognitive or colo#r or compassion* or coping or couples or dance or depression or directive or exercise or family or gestalt) W2 (therap* or treatment or train* or retrain* or rehabilitat* or adapt* or intervention or manag*) |
| S4 | ("acceptance commitment" or anxiety or art# or assertive or autosuggestion or aversive or behav*) W2 (therap* or treatment or train* or retrain* or rehabilitat* or adapt* or intervention or manag*) |
| S3 | (adjustment or attention or confidence or "day to day" or loss or physical or reality or suggestion) W1 (therap* or treatment or train* or retrain* or rehabilitat* or adapt* or intervention or manag*) |
| S2 | "eye movement" W2 (desensiti?ation or reprocessing) |
| S1 | (MH "Psychotherapy+") OR (MH "Rehabilitation, Psychosocial+") OR (MH "Cognitive Therapy+") |
|  |  |
|  |  |

**Table S2: IRIS Search strategies and output**

|  | psychotherapy and Subject: contains female circumcision |
| --- | --- |
|  | cognitive therapy and Subject: contains female circumcision |

**Table S3: Medline Search strategies and output**

|  | 1. exp Adaptation, Psychological/  2. exp Conditioning, Psychological/ or exp conditioning, classical/ or exp conditioning, operant/  3. exp Counseling/ or exp Dyssomnias/pc, th  4. exp Exercise Therapy/  5. (Eye Movement adj2 (Desensitiation or Reprocessing)).mp.  6. exp Mind-Body Therapies/  7. exp psychotherapy/  8. exp Sensory Art Therapies/ or Sleep/th or exp Sleep Hygiene/ or exp systems theory/  9. ((adjustment or attention or confidence or "day to day" or loss or physical or reality or suggestion) adj1 (therap* or treatment* or train* or retrain* or rehabilitat* or adapt* or intervention* or manag*)).mp.  10. ((Acceptance commitment or anxiety or Art? or Assertive or autosuggestion or Aversive or Behav*) adj2 (therap* or treatment* or train* or retrain* or rehabilitat* or adapt* or intervention* or manag*)).mp.  11. ((Client cent* or Cognitive or Colo?r or Compassion* or coping or couples or dance or depression or Directive or Exercise or Family or gestalt) adj2 (therap* or treatment* or train* or retrain* or rehabilitat* or adapt* or intervention* or manag*)).mp.  12. ((Human Givens or Humanistic or implosive or Interpersonal or language or marital or massage or memory or mentali?ation or milieu or music or narrative or nondirective or non-directive) adj2 (therap* or treatment* or train* or retrain* or rehabilitat* or adapt* or intervention* or manag*)).mp.  13. ((patient cent* or person cent* or play or psychoanalytic or rational emotive or reality or self or sleep or socioenvironmental or socio-environmental or stigma or suggestion or systemic or systems or therapeutic community) adj2 (therap* or treatment* or train* or retrain* or rehabilitat* or adapt* or intervention* or manag*)).mp.  14. (relax* adj3 (therap* or intervention* or treat*)).mp.  15. (abreaction or aromatherap* or asanas or autogenic train* or Behav* modification or bibliotherap* or (biofeedback adj2 psychol*)).mp.  16. (catharsis or classical conditioning or Compassionate Mind Train* or (conditioning adj2 psychol*) or counsel?ing or crisis intervention).mp.  17. ((Desensiti?ation adj2 psychol*) or Emotional freedom tapping or (feedback adj2 psychol*) or Flooding or free association or hypnosis or hypnotherapy).mp.  18. (imagery or logotherapy or meditation or Mindfulness or motivational interview* or mozart* or music* or neurofeedback or nonpharmacol* or "non-pharmacol*" or operant conditioning).mp.  19. (pranayama or progressive muscle relax* or psychodrama or Psychological Adaptation or psychological feedback or psychotherap*).mp.  20. (residential treatment? or Rewind technique? or sensory feedback or Stress manag* or transactional analysis or yoga).mp.  21. or/1-20  22. exp Circumcision, Female/  23. ((female or girl or girls or women or woman) adj3 (circumcis* or genital cut* or genital mutilat*)).mp.  24. 22 or 23  25. 21 and 24  26. remove duplicates from 25 |
| --- | --- |

**Table S4: PsycINFO Search strategies and output**

| S23 | S17 AND S22 |
| --- | --- |
| S22 | S20 OR S21 |
| S21 | (female or girl or girls or women or woman) N3 (circumcis* or "genital cut*" or "genital mutilat*" |
| S20 | S18 AND S19 |
| S19 | (DE "Female Genitalia") OR (DE "Human Females") |
| S18 | DE "Circumcision" |
| S17 | S1 OR S2 OR S3 OR S4 OR S5 OR S6 OR S7 OR S8 OR S9 OR S10 OR S11 OR S12 OR S13 OR S14 OR S15 OR S16 |
| S16 | "residential treatment" or "rewind technique" or "sensory feedback" or "stress manag*" or "transactional analysis" or yoga |
| S15 | imagery or logotherapy or meditation or mindfulness or "motivational interview*" or mozart* or music* or neurofeedback or nonpharmacol* or "non-pharmacol*" or "operant conditioning" or pranayama or "progressive muscle relax*" or psychodrama or "psychological adaptation" or "psychological feedback" or psychotherap* |
| S14 | (desensiti?ation N2 psychol*) or "emotional freedom tapping" or (feedback N2 psychol*) or flooding or "free association" or hypnosis or hypnotherapy |
| S13 | catharsis or "classical conditioning" or "compassionate mind train*" or (conditioning N2 psychol*) or counsel#ing or "crisis intervention" |
| S12 | abreaction or aromatherap* or asanas or "autogenic train*" or "behav* modification" or bibliotherap* or (biofeedback N2 psychol*) |
| S11 | relax* W3 (therap* or intervention or treat*) |
| S10 | ("patient cent*" or "person cent*" or play or psychoanalytic or "rational emotive" or reality or self or sleep or socioenvironmental or "socio-environmental" or stigma or suggestion or systemic or systems or "therapeutic community") W2 (therap* or treatment or train* or retrain* or rehabilitat* or adapt* or intervention or manag*) |
| S9 | ("Human Givens" or humanistic or implosive or Interpersonal or language or marital or massage or memory or mentali?ation or milieu or music or narrative or nondirective or "non-directive") W2 (therap* or treatment or train* or retrain* or rehabilitat* or adapt* or intervention or manag*) |
| S8 | ("client cent*" or cognitive or colo#r or compassion* or coping or couples or dance or depression or directive or exercise or family or gestalt) W2 (therap* or treatment or train* or retrain* or rehabilitat* or adapt* or intervention or manag*) |
| S7 | ("acceptance commitment" or anxiety or art# or assertive or autosuggestion or aversive or behav*) W2 (therap* or treatment or train* or retrain* or rehabilitat* or adapt* or intervention or manag*) |
| S6 | (adjustment or attention or confidence or "day to day" or loss or physical or reality or suggestion) W1 (therap* or treatment or train* or retrain* or rehabilitat* or adapt* or intervention or manag*) |
| S5 | "eye movement" W2 (desensiti?ation or reprocessing) |
| S4 | DE "Cognitive Behavior Therapy" OR DE "Acceptance and Commitment Therapy" OR DE "Cognitive Processing Therapy" OR DE "Prolonged Exposure Therapy" |
| S3 | DE "Behavior Therapy" OR DE "Aversion Therapy" OR DE "Covert Sensitization" OR DE "Conversion Therapy" OR DE "Dialectical Behavior Therapy" OR DE "Exposure Therapy" OR DE "Imaginal Exposure" OR DE "Implosive Therapy" OR DE "In Vivo Exposure" OR DE "Prolonged Exposure Therapy" OR DE "Systematic Desensitization Therapy" OR DE "Virtual Reality Exposure Therapy" OR DE "Implosive Therapy" OR DE "Reciprocal Inhibition Therapy" OR DE "Response Cost" OR DE "Systematic Desensitization Therapy" |
| S2 | DE "Psychosocial Rehabilitation" OR DE "Psychosocial Readjustment" OR DE "Therapeutic Social Clubs" OR DE "Vocational Rehabilitation" OR DE "Supported Employment" OR DE "Vocational Evaluation" OR DE "Work Adjustment Training" |
| S1 | DE "Psychotherapy" OR DE "Adolescent Psychotherapy" OR DE "Multisystemic Therapy" OR DE "Child Psychotherapy" OR DE "Play Therapy" OR DE "Gestalt Therapy" OR DE "Empty Chair Technique" OR DE "Group Psychotherapy" OR DE "Encounter Group Therapy" OR DE "Therapeutic Community" OR DE "Humanistic Psychotherapy" OR DE "Client Centered Therapy" OR DE "Hypnotherapy" OR DE "Age Regression (Hypnotic)" OR DE "Ericksonian Psychotherapy" OR DE "Posthypnotic Suggestions" OR DE "Integrative Psychotherapy" OR DE "Schema Therapy" OR DE "Psychoanalysis" OR DE "Adlerian Psychotherapy" OR DE "Brief Relational Therapy" OR DE "Dream Analysis" OR DE "Self-Analysis" OR DE "Psychotherapeutic Counseling" OR DE "Family Therapy" OR DE "Psychotherapeutic Techniques" OR DE "Active Listening" OR DE "Animal Assisted Therapy" OR DE "Autogenic Training" OR DE "Brief Relational Therapy" OR DE "Centering" OR DE "Cotherapy" OR DE "Dream Analysis" OR DE "Empty Chair Technique" OR DE "Ericksonian Psychotherapy" OR DE "Free Association" OR DE "Guided Imagery" OR DE "Life Review" OR DE "Mirroring" OR DE "Morita Therapy" OR DE "Motivational Interviewing" OR DE "Mutual Storytelling Technique" OR DE "Network Therapy" OR DE "Paradoxical Techniques" OR DE "Psychodrama" OR DE "Self-Affirmation" OR DE "Strategic Therapy" OR DE "Strategic Family Therapy" OR DE "Marriage Counseling" OR DE "Conjoint Therapy" OR DE "Medication-Assisted Treatment" OR DE "Methadone Maintenance" OR DE "Psychotherapeutic Processes" OR DE "Contemporaneity" OR DE "Countertransference" OR DE "Enactments" OR DE "Insight (Psychotherapeutic Process)" OR DE "Negative Therapeutic Reaction" OR DE "Psychotherapeutic Breakthrough" OR DE "Psychotherapeutic Neutrality" OR DE "Psychotherapeutic Resistance" OR DE "Psychotherapeutic Transference" OR DE "Therapeutic Alliance" |

**Table S5: SCOPUS Search strategies and output**

SCOPUS

|  | ((TITLE-ABS-KEY("residential treatment" or "rewind technique" or "sensory feedback" or "stress manag*" or "transactional analysis" or yoga)) OR (TITLE-ABS-KEY(pranayama or "progressive muscle relax*" or psychodrama or "psychological adaptation" or "psychological feedback" or psychotherap*)) OR (TITLE-ABS-KEY(imagery or logotherapy or meditation or mindfulness or "motivational interview*" or mozart* or music* or neurofeedback or nonpharmacol* or non-pharmacol* or "operant conditioning")) OR (TITLE-ABS-KEY((feedback W/2 psychol*) or flooding or "free association" or hypnosis or hypnotherapy)) OR (TITLE-ABS-KEY((desensiti?ation W/2 psychol*) or "emotional freedom tapping" or ("eye movement" W/2 (desensiti?ation or reprocessing)))) OR (TITLE-ABS-KEY(catharsis or "classical conditioning" or "Compassionate Mind Train*" or (conditioning W/2 psychol*) or counseling or counselling or "crisis intervention")) OR (TITLE-ABS-KEY(abreaction or aromatherap* or asanas or "autogenic train*" or "behav* modification" or bibliotherap* or (biofeedback W/2 psychol*))) OR (TITLE-ABS-KEY(relax* W/3 (therap* or intervention* or treat*))) OR (TITLE-ABS-KEY(("patient cent*" or "person cent*" or play or psychoanalytic or "rational emotive" or reality or self or sleep or socioenvironmental or socio-environmental or stigma or suggestion or systemic or systems or "therapeutic community") W/2 (therap* or treatment* or train* or retrain* or rehabilitat* or adapt* or intervention* or manag*))) OR (TITLE-ABS-KEY(("Human Givens" or humanistic or implosive or Interpersonal or language or marital or massage or memory or mentali?ation or milieu or music or narrative or nondirective or non-directive) W/2 (therap* or treatment* or train* or retrain* or rehabilitat* or adapt* or intervention* or manag*))) OR (TITLE-ABS-KEY(("client cent*" or cognitive or colour or color or compassion* or coping or couples or dance or depression or directive or exercise or family or gestalt) W/2 (therap* or treatment* or train* or retrain* or rehabilitat* or adapt* or intervention* or manag*))) OR (TITLE-ABS-KEY(("acceptance commitment" or anxiety or art or arts or assertive or autosuggestion or aversive or behav*) W/2 (therap* or treatment* or train* or retrain* or rehabilitat* or adapt* or intervention* or manag*))) OR (TITLE-ABS-KEY((adjustment or attention or confidence or "day to day" or loss or physical or reality or suggestion) W/1 (therap* or treatment* or train* or retrain* or rehabilitat* or adapt* or intervention* or manag*)))) AND (TITLE-ABS-KEY((female or girl or girls or women or woman) W/3 (circumcis* or "genital cut*" or "genital mutilat*"))) |
| --- | --- |

**Table S6: Web of Science Search strategies and output**

|  | 1: TS=("residential treatment$" or "Rewind technique$" or "sensory feedback" or "stress manag*" or "transactional analysis" or yoga)  2: TS=(pranayama or "progressive muscle relax*" or psychodrama or "psychological adaptation" or "psychological feedback" or psychotherap*)  3: TS=(imagery or logotherapy or meditation or Mindfulness or "motivational interview*" or mozart* or music* or neurofeedback or nonpharmacol* or "non-pharmacol*" or "operant conditioning")  4: TS=((Desensiti$ation NEAR/2 psychol*) or "Emotional freedom tapping" or (feedback NEAR/2 psychol*) or Flooding or "free association" or hypnosis or hypnotherapy)  5: TS=(catharsis or "classical conditioning" or "Compassionate Mind Train*" or (conditioning NEAR/2 psychol*) or counsel$ing or crisis intervention)  6: TS=(abreaction or aromatherap* or asanas or "autogenic train*" or "Behav* modification" or bibliotherap* or (biofeedback NEAR/2 psychol*))  7: TS=(relax* NEAR/3 (therap* or intervention* or treat*))  8: TS=(("patient cent*" or "person cent*" or play or psychoanalytic or "rational emotive" or reality or self or sleep or socioenvironmental or "socio-environmental" or stigma or suggestion or systemic or systems or "therapeutic community") NEAR/2 (therap* or treatment* or train* or retrain* or rehabilitat* or adapt* or intervention* or manag*))  9: TS=((adjustment or attention or confidence or "day to day" or loss or physical or reality or suggestion) NEAR/1 (therap* or treatment* or train* or retrain* or rehabilitat* or adapt* or intervention* or manag*))  10: TS=(("acceptance commitment" or anxiety or art$ or assertive or autosuggestion or aversive or behav*) NEAR/2 (therap* or treatment* or train* or retrain* or rehabilitat* or adapt* or intervention* or manag*))  11: TS=(("Client cent*" or Cognitive or Colo$r or Compassion* or coping or couples or dance or depression or Directive or Exercise or Family or gestalt) NEAR/2 (therap* or treatment* or train* or retrain* or rehabilitat* or adapt* or intervention* or manag*))  12: TS=(("Human Givens" or Humanistic or implosive or Interpersonal or language or marital or massage or memory or mentali$ation or milieu or music or narrative or nondirective or "non-directive") NEAR/2 (therap* or treatment* or train* or retrain* or rehabilitat* or adapt* or intervention* or manag*))  13: #1 OR #2 OR #3 OR #4 OR #5 OR #6 OR #7 OR #8 OR #9 OR #10 OR #11 OR #12  14: (female or girl or girls or women or woman) NEAR/3 (circumcis* or "genital cut*" or "genital mutilat*") (Topic)  15: #13 AND #14 |
| --- | --- |

**Additional Search strategies (01/01/2023 – 11/08/2025)**

**MEDLINE & CINAHL (17 Hits)**

("female genital mutilation" or FGM or "female circumcision" or infibulation or clitoridectomy or excision or "Type I FGM" or "Type II FGM" or "Type III FGM" or "Type IV FGM") AND ("sexual dysfunction" or "sexual health" or "sexual function" or "female sexual dysfunction" or "sexual desire disorder" or arousal or orgasm or dyspareunia or "sexual satisfaction" or libido) AND (counseling or "psychosexual therapy" or psychotherapy or "physical therapy" or "pelvic floor muscle exercise" or rehabilitation or "sexual education" or "behavioral therapy" or "sex therapy" or "mechanical device" or lubricants or "non-surgical intervention")

**PMC (3 Hits)**

(("Genital Mutilation, Female"[MeSH] OR "female genital mutilation"[tiab] OR FGM[tiab] OR "female circumcision"[tiab] OR infibulation[tiab] OR clitoridectomy[tiab] OR excision[tiab] OR "Type I FGM"[tiab] OR "Type II FGM"[tiab] OR "Type III FGM"[tiab] OR "Type IV FGM"[tiab])

AND

("Sexual Dysfunction, Physiological"[MeSH] OR "Sexual Dysfunctions, Psychological"[MeSH] OR "sexual dysfunction"[tiab] OR "sexual health"[tiab] OR "sexual function"[tiab] OR "female sexual dysfunction"[tiab] OR "sexual desire disorder"[tiab] OR "arousal disorder"[tiab] OR "orgasm disorder"[tiab] OR dyspareunia[tiab] OR "sexual satisfaction"[tiab] OR libido[tiab])

AND

("Counseling"[MeSH] OR "Psychotherapy"[MeSH] OR "Rehabilitation"[MeSH] OR "Physical Therapy Modalities"[MeSH] OR "Behavior Therapy"[MeSH]

OR counseling[tiab] OR "psychosexual therapy"[tiab] OR psychotherapy[tiab] OR "physical therapy"[tiab] OR "pelvic floor muscle exercise"[tiab] OR rehabilitation[tiab]

OR "sexual education"[tiab] OR "behavioral therapy"[tiab] OR "sex therapy"[tiab] OR "mechanical device"[tiab] OR lubricants[tiab] OR "non-surgical intervention"[tiab]))
