# Supplementary material for: Sexual health interventions for treating sexual dysfunction in women with female genital mutilation: A systematic review
Source: Int J Gynaecol Obstet. 2026 Jan 26;172(Suppl 1):9–19. doi: 10.1002/ijgo.70761 (PMC12833628; doi:10.1002/ijgo.70761)
Supplement: Supplementary file 2 — File S2. [file IJGO-172-9-s002.docx]

**Supplementary File 2**

**References for Excluded Studies**

Abdulcadir J, Bianchi Demicheli F, Willame A, Recordon N, Petignat P. Posttraumatic stress disorder relapse and clitoral reconstruction after female genital mutilation. Obstetrics & Gynecology. 2017;129(2):371-6.

Beltran L, Fall S, Antonetti-N'Diaye E. Excision: entre clinique et droits humains. Sexologies: European Journal of Sexology and Sexual Health / Revue européenne de sexologie et de santé sexuelle. 2015;24(3):122-7.

Elnashar AM, El-Dien Ibrahim M, El-Desoky MM, Ali OM, El-Sayd Mohamed Hassan M. Female sexual dysfunction in Lower Egypt. BJOG: An International Journal of Obstetrics & Gynaecology. 2007;114(2):201-6.

Ezebialu I, Okafo O, Oringanje C, Ogbonna U, Udoh E, Odey F, Meremikwu MM. Surgical and nonsurgical interventions for vulvar and clitoral pain in girls and women living with female genital mutilation: a systematic review. International Journal of Gynaecology & Obstetrics. 2017;136(Suppl 1):34-7.

Foldes P, Droupy S, Cuzin B. Cosmetic surgery of the female genitalia. Progres En Urologie. 2013;23(9):601-11. doi: 10.1016/j.purol.2013.01.017.

Mahgoub E, Nimir M, Abdalla S, Elhuda DA. Effects of school-based health education on attitudes of female students towards female genital mutilation in Sudan. Eastern Mediterranean Health Journal. 2019;25(6):406-12. doi: 10.26719/emhj.18.05.

Okomo U, Ogugbue M, Inyang E, Meremikwu MM. Sexual counselling for treating or preventing sexual dysfunction in women living with female genital mutilation: a systematic review. International Journal of Gynaecology & Obstetrics. 2017;136(Suppl 1):38-42. doi: 10.1002/ijgo.12049.

Villani M. Reconstructing sexuality after excision: the medical tools. Medical Anthropology. 2020;39(3):269-81. doi: 10.1080/01459740.2019.1665670.
